# Mixed Lymphocyte Reaction: Functional Immune Profiling in Transplantation and Beyond

**DOI:** 10.3390/diagnostics16060929

**Published:** 2026-03-20

**Authors:** Nurtilek Galimov, Aruzhan Asanova, Sholpan Altynova, Aidos Bolatov

**Affiliations:** 1 Department of Science, “University Medical Center” Corporate Fund, Astana 010000, Kazakhstan; nurtilek.galimov@nu.edu.kz (N.G.); asanova.aruzhan@umc.org.kz (A.A.); 2 School Sciences and Humanities, Nazarbayev University, Astana 010000, Kazakhstan; 3 Department of Medical and Regulatory Affairs, “University Medical Center” Corporate Fund, Astana 010000, Kazakhstan; venera.altynova@umc.org.kz; 4 Shenzhen University Medical School, Shenzhen University, Shenzhen 518060, China

**Keywords:** mixed lymphocyte culture, alloreactivity, allorecognition, transplantation immunology, cancer immunotherapy, biomarkers, personalized medicine

## Abstract

The mixed lymphocyte reaction (MLR) is a classic functional assay that models in vitro interactions between responder T cells and allogeneic antigen-presenting cells (APCs). It quantifies the magnitude and quality of alloreactivity, integrating signals from allorecognition, co-stimulation, inflammatory context, and minor histocompatibility antigens that may not be captured by molecular matching alone. This review is narrative in nature and is intended as a practical, non-systematic synthesis of the field. To provide a modern, practice-oriented synthesis of MLR designs, readouts, and translational uses, highlighting how new technologies have expanded MLR from bulk proliferation into multidimensional immune profiling.We summarize why MLR remains valuable as a functional compatibility probe beyond HLA typing, including the high baseline frequency of alloreactive T cells that produces robust signals without priming. We then review key design options (one-way vs. two-way formats; stimulator inactivation; responder definition; APC source and maturation) and how these choices affect interpretation for rejection and graft-versus-host disease risk modeling, tolerance-focused studies, and immunomodulatory screening. Next, we outline major readouts—radiometric and flow cytometric proliferation (dye dilution, Ki-67), cytokine/chemokine profiling, cytotoxicity adaptations, and next-generation add-ons (e.g., scRNA-seq, TCR sequencing)—emphasizing complementary strengths and common pitfalls. Finally, we consolidate practical quality and reproducibility controls (donor variability, dynamic range, timing, batch effects, and acceptance criteria) to improve cross-study comparability and translational readiness. Modern MLR platforms combine controllable allogeneic stimulation with scalable, high-resolution readouts for mechanistic discovery, immune monitoring and translational immune profiling. Standardized modular design and rigorous quality control can improve reproducibility and support broader adoption across transplantation, immunotherapy, and immune-modulation research.

## 1. Introduction

The immune system is an extremely complex network of cells, tissues and organs that protects the host from pathogens while maintaining tolerance to self. This dynamic balance is mediated by innate and adaptive immune responses, the latter providing specificity, memory and durable protection. The key player in adaptive immunity is the activation of T lymphocytes, which recognize antigens via T-cell receptors (TCRs) presented on major histocompatibility complex (MHC) molecules by antigen-presenting cells (APCs).

The mixed lymphocyte reaction (MLR)—also termed the mixed lymphocyte culture (MLC)—is one of the most enduring functional assays in immunology, designed to recreate in vitro the core cellular event that underlies allogeneic transplantation: the encounter between responder T cells and allogeneic APCs from a genetically distinct individual [1,2]. In its classic form, the assay quantifies both the magnitude and the functional quality of alloreactivity. This response is typically intense because responder T cells recognize non-self MHC determinants (HLA in humans) and other alloantigens on donor cells. The ensuing reaction is marked by T-cell activation, blast transformation, entry into DNA synthesis, and progressive clonal expansion through proliferation [3,4,5]. First described as a striking proliferative phenomenon when leukocytes from unrelated individuals were co-cultured (1961–1964) [6,7,8,9], the MLR rapidly became a practical “biological probe” for histocompatibility and immune function long before the molecular architecture of HLA was fully resolved.

As a functional assay of T-cell alloreactivity, MLR models the cellular interactions that shape transplantation immunity. In its most common implementation, responder lymphocytes are co-cultured with allogeneic stimulator APCs, typically within peripheral blood mononuclear cell (PBMC)-derived preparations, to quantify proliferative and functional responses to non-self MHC. CD4^+^ T cells primarily recognize alloantigens presented on MHC class II and coordinate downstream immune polarization, whereas CD8^+^ cytotoxic T lymphocytes respond to class I-associated signals and contribute to graft injury; natural killer (NK) cells may also modulate the response in selected settings [10,11,12]. Stimulator potency depends strongly on APC composition, with dendritic-cell subsets often providing the strongest activation signals because of their high MHC and co-stimulatory molecule expression [13].

A defining strength of the MLR is that it interrogates function, not merely genotype. While contemporary transplantation relies heavily on high-resolution HLA typing and donor-specific antibody assessment, clinical outcomes are still shaped by the integrated effect of direct and indirect allorecognition, co-stimulation, inflammatory context, and the contribution of minor histocompatibility antigens (miHAs). The MLR provides a controlled platform that approximates the donor–recipient cellular interface and can expose immunological risk that is not always inferable from molecular matching alone. Historically, this functional perspective helped establish MLR as a practical complement to molecular compatibility assessment [3,4].

Methodologically, the assay is commonly implemented in two principal formats with distinct interpretive value. In one-way MLR, one cell population (stimulators) is rendered non-proliferative, classically by mitomycin C treatment or irradiation, so that measured proliferation arises predominantly from the responder compartment, enabling directional assessment of recipient anti-donor reactivity (rejection risk) or donor anti-recipient reactivity (graft-versus-host disease risk). The one-way approach was formalized in the mid-1960s and remains foundational for translational designs [14,15,16,17]. In two-way MLR, both populations remain capable of proliferating, producing a bidirectional response that provides a broader, less direction-specific summary of functional histocompatibility [14,18].

The historical trajectory of MLR mirrors the evolution of human immunogenetics. Early radiometric implementations used tritiated thymidine incorporation as a bulk surrogate for blastogenesis and DNA synthesis, enabling quantitative standardization and widespread adoption [19]. By the 1970s, genetic studies linked the strength of MLR to the HLA-D region, and subsequent work clarified that this “D region” encompassed multiple class II sub-loci (HLA-DR, -DQ, -DP), embedding MLR within the emerging framework of HLA class II-restricted alloreactivity [20,21]. These developments established the biological and genetic foundations of the assay, but the major shift in its contemporary relevance has come from newer analytical technologies.

Over the last three decades, MLR has been reinvented by technologies that shift the assay from a single bulk number into a multidimensional immune profile. A pivotal advance was the introduction of dye-dilution flow cytometry methods for division tracking (notably carboxyfluorescein succinimidyl ester, CFSE), which enabled resolution of proliferative history at the single-cell level while simultaneously identifying phenotypes such as CD4^+^ versus CD8^+^ T cells and linking proliferation to activation or effector functions [22,23]. This multiparametric paradigm has since expanded into high-throughput flow platforms supporting 96-/384-well formats and combined readouts (proliferation, viability, cytokines, surface activation markers), positioning MLR as a scalable screening tool for immunosuppressive and immunomodulatory drug development [24]. In parallel, repertoire-level approaches now use MLR as an ex vivo “enrichment step” to define and longitudinally track donor-reactive T-cell clones via TCR sequencing, creating individualized alloresponse fingerprints with direct relevance to immune monitoring after transplantation [25,26].

Given this modernization, the contemporary MLR is best viewed not as a legacy assay but as a flexible experimental ecosystem: a controllable allogeneic stimulus paired with increasingly precise readouts that bridge mechanistic immunology and clinical translation. However, despite this evolution, MLR is still often discussed either as a historical compatibility assay or in highly specialized methodological contexts, and the field lacks a concise integrative overview linking assay design, readout selection, quality considerations, and translational application. This creates a practical gap for investigators seeking to deploy MLR in modern settings such as immune monitoring, immunosuppressive drug evaluation, cellular therapy, and biomarker-guided translational research.

The aim of this review is therefore to provide a conceptually integrated and practice-oriented synthesis of contemporary MLR methodology and relevance. This article is intended as a narrative review. The literature discussed was selected to provide a conceptually integrated, practice-oriented overview of the evolution of mixed lymphocyte reaction (MLR) methods, readouts, quality considerations, and translational applications. In this review, we synthesize (i) current MLR designs and sources of technical variability; (ii) classical and next-generation readouts spanning radiometric, flow cytometric, multiplex and sequencing-based approaches; and (iii) translational applications across transplantation immunology, immunosuppressive drug evaluation, cellular therapy, and emerging tolerance- and immunotherapy-oriented strategies. By addressing the gap between classical assay concepts and current multiparametric, clinically relevant implementations, we aim to provide a practical framework for using MLR as a rigorous, high-resolution tool in contemporary immunological research and translational medicine.

## 2. Why MLR Still Matters: Functional Compatibility Beyond HLA Typing

**Figure 1:**
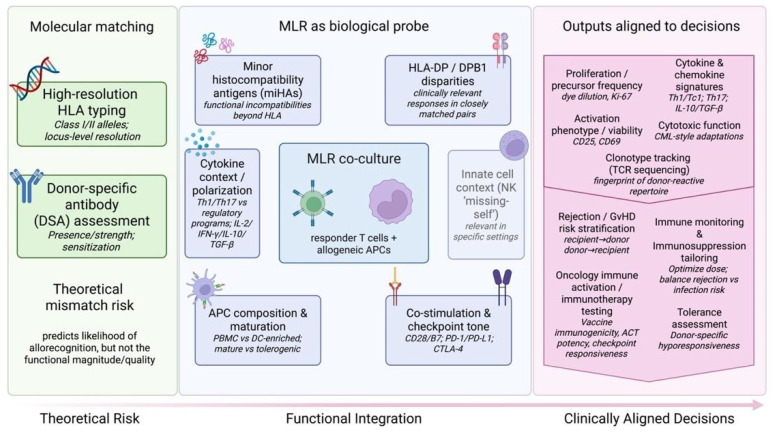
“Functional bridge” concept map: from genotype to biological response. Left: high-resolution human leukocyte antigen (HLA) typing and donor-specific antibody (DSA) assessment estimate theoretical mismatch risk. Center: the mixed lymphocyte reaction (MLR) functions as a biological probe integrating antigenic and contextual drivers of alloreactivity that may be under-resolved by molecular matching alone, including minor histocompatibility antigens (miHAs), HLA-DP/DPB1 disparities, antigen-presenting cell (APC) composition and maturation state, co-stimulation/checkpoint tone, cytokine polarization, and (in selected settings) innate “missing-self” effects. Right: multiparametric outputs (proliferation/precursor frequency, activation phenotype/viability, cytokine/chemokine signatures, cytotoxicity, and optional T-cell receptor (TCR) clonotype tracking) align MLR readouts with translational decisions spanning rejection/graft-versus-host disease (GvHD) risk stratification, immune monitoring and immunosuppression tailoring, tolerance assessment, and oncology-oriented immune activation testing. Created with BioRender.com.

While high-resolution HLA typing is the primary method for donor–recipient matching, the MLR still offers a functional assessment of immunological compatibility that complements genetic typing [4]. In this context, molecular matching can be viewed as a largely theoretical measure of compatibility, whereas MLR serves as a biological probe (an in vitro model) that more directly simulates the immunological interaction between donor and recipient (Figure 1) [27,28]. This functional design also helps explain why MLR can reveal clinically meaningful incompatibilities that are not fully captured by standard typing approaches. For example, MLR can detect alloreaction to MHC or conformational epitopes that are not easily identified by HLA typing alone, and it may uncover “hidden” mismatches driven by miHAs or underappreciated differences in loci such as HLA-DP [29]. Because it measures the actual activation and expansion of responder T cells against “non-self” APCs, the assay has historically been viewed as a final arbiter of compatibility in settings where biological consequences matter more than genotype alone [30,31].

Beyond baseline compatibility assessment, MLR is used for immune monitoring, where tracking the strength and quality of T-cell responses over time can provide insight into rejection risk and the development of tolerance that molecular typing alone cannot provide [32]. Consistent with this direction, recent studies integrate MLR with high-throughput TCR sequencing to track donor-reactive T cells, supporting biomarker-oriented approaches for predicting rejection or tolerance [28]. In solid organ transplantation, MLR-based assessments of donor-specific hypo-responsiveness post-transplantation have been associated with better graft survival [33].

A second reason for the enduring relevance of MLR is the exceptionally large pool of alloreactive T cells, which generates a strong and readily quantifiable response. Due to the high frequency of alloreactive T cells, much greater than for pathogen-specific clones, robust proliferation and cytokine release can be induced in vitro [34]. This “inordinate” precursor frequency is often estimated at ~1–10% of the total T-cell repertoire, exceeding by more than an order of magnitude the typical precursor pool for conventional foreign antigens [15]. As a result, MLR can produce a robust measurable signal without prior sensitization (priming), supported by contributions from both naïve and memory alloreactive T cells [4]. The same property also underpins sensitivity to relatively subtle differences, including single miHAs loci, enabling a high-specificity functional “fingerprint” of donor-reactive immunity [35].

## 3. MLR Assay Design and Variants

A central design choice in the MLR is whether one or both cellular compartments are allowed to respond to allogeneic stimulation. In the two-way MLR, PBMCs from two genetically distinct individuals are mixed so that each population simultaneously serves as both responder and stimulator. Because both partners display allogeneic MHC determinants that can be recognized as non-self, both undergo blast transformation, DNA synthesis, and proliferation, generating a bidirectional composite signal. This format provides a global measure of functional histocompatibility, but it does not distinguish how much each individual contributes to the net response [34].

In contrast, the one-way MLR is designed to isolate a single direction of alloreactivity by rendering one population (the stimulators) mitotically inactive while preserving antigen presentation. Proliferation and downstream readouts then arise predominantly from the responders, enabling a directional interpretation that is directly relevant for translational questions [4,36]. Directionality is particularly important for modeling distinct clinical risks: (i) rejection risk is approximated by combining recipient responder cells with inactivated donor stimulators, whereas (ii) graft-versus-host disease (GvHD) risk is approximated by combining donor responders with inactivated recipient stimulators [14,37,38,39]. Because it restricts the biological signal to a defined responding compartment, one-way MLR is generally preferred for mechanistic and clinical-aligned study designs [4].

To establish a one-way reaction, stimulator cells must be prevented from dividing without destroying their capacity to trigger responder activation. Two widely used approaches are: irradiation (commonly in the ~20–30 Gy range in many protocols) and mitomycin C (MMC) exposure followed by extensive washing. Peer-reviewed protocols frequently use ~30 Gy irradiation for stimulator PBMCs (example implementation in a human MLC) [40]. Likewise, MMC-based one-way MLR protocols commonly report conditions around 50 μg/mL for ~30 min at 37 °C, with repeated washes to remove residual drug [41,42].

Table 1 summarizes the main cellular design choices in MLR, including responder definition, APC source, and maturation state, together with their main interpretive trade-offs. Rather than representing interchangeable formats, these configurations shape whether the assay is best suited to global compatibility testing, directional alloresponse modeling, or tolerance- and immune-modulation studies (Table 1).

Overall, MLR design is best understood as a modular framework in which directionality, stimulator inactivation, responder definition, and APC composition jointly determine the biological meaning of the readout. These parameters influence whether the assay captures a global compatibility phenotype, a directional clinically interpretable alloresponse, or a more mechanistically tuned immune program.

Table 2 summarizes the principal experimental approaches used to quantify MLR outcomes, from classical bulk proliferation assays to multiparametric and repertoire-level profiling methods. Modern studies increasingly combine these readout classes because they capture complementary layers of alloreactivity: bulk proliferation reflects response magnitude, flow cytometry resolves responding subsets and activation states, cytokine-based assays define functional polarization, and sequencing-based approaches provide clonotypic or systems-level resolution [25,26,47,48,49,50,51,52,53,54,55,56,57,58].

Accordingly, readout selection should be driven by the biological question and required level of resolution, rather than by technical convention alone. In practice, combining orthogonal readouts often provides a more informative view of allostimulation than any single platform in isolation.

## 4. Outcome Readouts: From Bulk Proliferation to Next-Generation Immune Profiling

Assessment of MLR outcomes has evolved substantially, moving from bulk measures of proliferation toward subset-resolved and multiparametric immune profiling. Traditional ^3^H-thymidine incorporation remains a sensitive benchmark for DNA synthesis, but it does not identify which cellular lineages are proliferating. This limitation contributed to the widespread adoption of flow cytometry-based dye dilution approaches (e.g., CFSE and related tracers), which quantify division history at single-cell resolution and can be integrated with activation and viability markers. Ki-67 staining provides an additional measure of proliferative activity, particularly when responses are subtle or dye dilution is suboptimal. The principal MLR readout classes and their interpretive value are summarized in Table 3.

Beyond proliferation, Table 3 highlights complementary readout layers that capture response quality, function, and mechanism. Multiplex cytokine/chemokine platforms help define inflammatory versus regulatory polarization, cytotoxicity-oriented adaptations measure effector capacity more directly, and next-generation approaches such as scRNA-seq and TCR sequencing add cell-state and clonotypic resolution to donor-reactive responses over time. Rather than serving as interchangeable outputs, these readouts provide distinct but complementary views of alloreactivity, and their selection should be driven by the biological question and the level of mechanistic resolution required.

## 5. Assay Quality, Reproducibility, and Standardization

Because MLR is a sample-dependent functional assay rather than a fully standardized quantitative test, reproducibility depends on controlling a limited set of variables: donor composition, responder-to-stimulator ratio, culture timing, batch effects, and predefined acceptance criteria. Table 4 summarizes these practical quality control (QC) domains and their rationale.

Taken together, these quality-control domains show that MLR reproducibility depends less on any single platform than on disciplined management of biological and analytical variability. Donor composition, responder-to-stimulator ratio, culture timing, batch control, and predefined acceptance criteria jointly determine whether an observed signal can be interpreted as robust and comparable rather than context-specific noise. Accordingly, standardization in MLR should be understood not as elimination of biological diversity, but as the use of controlled workflows that preserve interpretability across experiments, laboratories, and translational settings.

## 6. Applications in Transplantation

To provide a structured overview of how assay configuration and readout selection map to specific clinical questions in transplantation, the main MLR use-cases (compatibility assessment beyond HLA typing, rejection/GvHD risk modeling, immune monitoring/immunosuppression titration, and tolerance/mixed-chimerism frameworks) are summarized in Table 5.

While high-resolution HLA typing is the primary method for donor–recipient matching, the MLR still offers a functional assessment of immunological compatibility that complements genetic typing. It can detect alloreaction to major histocompatibility antigens or conformational epitopes which are not easily identified by HLA typing alone. The use of the MLR response correlates with the degree of HLA difference and the likelihood of allograft rejection [4]. Recent studies integrate MLR with high-throughput TCR-sequencing to track donor-reactive T-cells, supporting biomarker-oriented immune monitoring approaches for rejection and tolerance research [4,28]. In this way, MLR serves as a functional bridge between “theoretical” genetic matching and the real biological response, including incompatibilities driven by minor histocompatibility antigens (miHAs) and reactivity that may be under-recognized in standard matching workflows [3,73]. Reactivity mediated by the HLA-DP locus is a prominent example: DP-directed alloresponses can be detected in primary one-way MLR even in otherwise closely matched pairs, supporting the continued value of functional testing when DPB1 disparities are clinically relevant [78,79].

The MLR has been investigated as a functional assay for modeling alloreactivity relevant to GvHD and graft rejection in hematopoietic stem cell transplantation (HSCT) and solid organ transplant rejection. A strong MLR response between recipient T-cells and donor stimulator cells in a one-way MLR indicates high recipient alloreactivity, which is associated with an increased risk of graft rejection. While early studies on HLA-identical sibling bone marrow transplants showed limited correlation between MLR and acute GvHD severity, more recent investigations have explored the predictive value for chronic GvHD, with some studies suggesting a higher incidence of chronic GvHD in recipients with higher MLR reactivity [80]. Directionality in one-way MLR also enables risk modeling that aligns with clinical biology: recipient-versus-donor proliferation reflects rejection potential, whereas donor-versus-recipient proliferation reflects GvHD potential [14].

To enhance sensitivity in settings where baseline alloresponses are weak (e.g., HLA-identical siblings), modified MLC approaches have added exogenous cytokines (e.g., IL-2 with IL-4; or IL-2 with IFN-γ and TNF-α), and positive responses in these cytokine-enhanced formats have been investigated as predictors of GvHD [5,29,81]. For solid organ transplantation, MLR-based assessments of donor-specific hypo-responsiveness post-transplantation have been associated with better graft survival [33].

Mechanistically, efforts to improve prediction increasingly focus on responder subsets and cytokine signatures. The presence of regulatory T cells (Treg), often discussed alongside IL-17-associated immune patterns, has been linked to rejection biology across kidney, liver, and heart transplantation [82,83]. Elevated frequencies of CD4+ T-cells expressing pro-inflammatory cytokines like IFN-γ, IL-4, IL-6 and IL-17A in MLR, particularly in highly sensitized kidney transplant candidates, are indicative of heightened immune reactivity and a higher risk of antibody-mediated rejection and poor graft survival [11,84]. Cytokine release patterns in MLR, including IL-2, IL-4, IL-6 and IL-10, can also contribute to predicting anti-leukemic T-cell reactions and patient response to immunotherapy and GvHD [85,86]. The predictive value of MLR in clinical transplantation has been enhanced by integrating it with advanced techniques, such as flow cytometry-based assays and multiplex cytokine analysis, which allow for a more precise and comprehensive assessment of T-cell alloreactivity and functional outcomes [33]. Beyond bulk readouts, TCR repertoire approaches extend this concept by identifying donor-reactive clonotypes pre-transplant and tracking them longitudinally as signatures of rejection biology in blood and/or tissue [87].

The MLR is widely used to evaluate the efficacy of existing and novel immunosuppressive drugs, serving as a critical in vitro platform for optimizing therapeutic regimens and minimizing adverse effects. By titrating immunosuppressants into MLR cultures, researchers can determine the drug’s ability to inhibit T-cell proliferation, cytokine production and cytotoxic activity in a dose-dependent manner. This application is vital for optimizing drug dosages, identifying potential drug synergies and assessing the individual patient’s response to therapy, moving towards personalized immunosuppression [68].

Translationally, multiparametric CFSE-based MLR frameworks have been applied in living donor liver transplantation to quantify donor-reactive precursor frequencies and guide individualized immunosuppression intensity [81]. In prospective clinical experience, MLR-guided titration has been associated with reduced infectious complications and mortality compared with empirical dosing strategies [81,88].

Current evidence supports MLR as a complementary functional assay that should be interpreted alongside routine clinical and biomarker data, rather than as a stand-alone trigger for immunosuppression adjustment. The strongest direct human evidence comes from liver transplantation: Tanaka et al. (2012) used a multiparametric CFSE-based MLR to guide immunosuppressive therapy and reported fewer infectious complications and lower mortality than with empirical dosing, although this was a single-center study and did not define a universal workflow [81]. Sakai et al. (2014) similarly reported favorable outcomes with CFSE-MLR–based monitoring in selected liver-transplant recipients, supporting the use of MLR as a functional immune-status readout to help individualize management in specific settings [89].

More broadly, transplant immune-monitoring reviews emphasize that no single assay is sufficient in isolation and that functional assays should be interpreted together with graft function, drug exposure, donor-specific antibodies (DSA), donor-derived cell-free DNA (dd-cfDNA), biopsy context, and infection history [90,91,92,93]. Recipient stratification may also affect interpretability: for example, Iwahara et al. (2023) showed that donor-specific hyporesponsiveness differed between DSA-positive and DSA-negative kidney transplant recipients, suggesting that MLR-type assays are most informative when embedded in a broader risk-stratification framework [94].

However, standardized implementation pathways for serial post-transplant MLR monitoring are still lacking. We did not identify a peer-reviewed guideline or consensus statement defining a routine early post-transplant MLR schedule, and current reviews support serial monitoring in principle without specifying an MLR-specific regimen. Accordingly, MLR-guided monitoring should presently be viewed as a promising translational strategy that still requires prospective validation, assay harmonization, and clearer workflow standardization before routine implementation [90,91].

**Figure 2:**
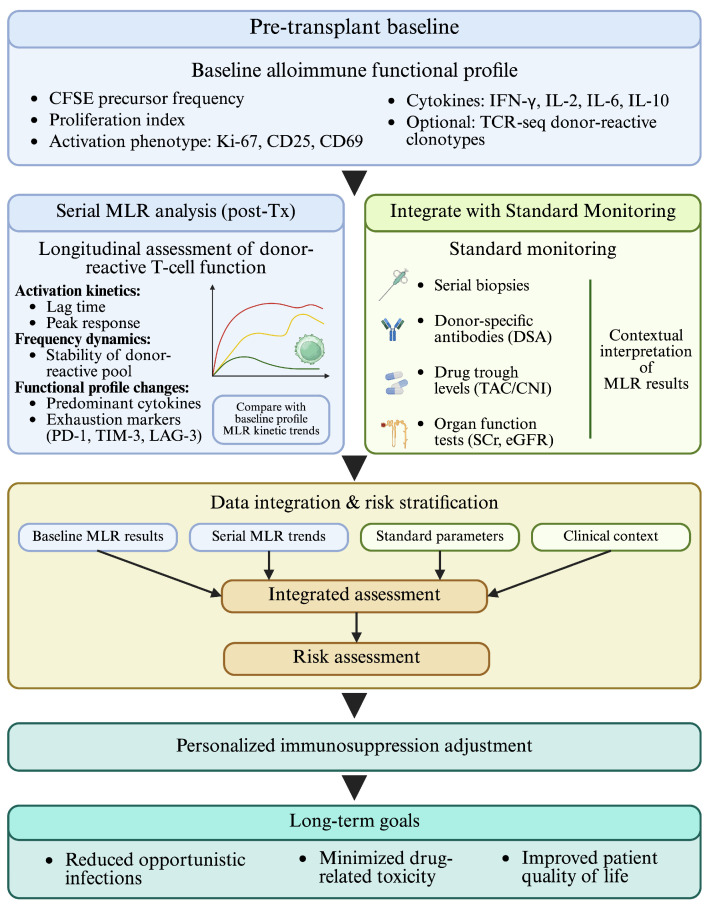
Conceptual framework for integrating mixed lymphocyte reaction (MLR) into post-transplant monitoring and personalized immunosuppression adjustment. Created with BioRender.com. This figure is intended as a conceptual translational model; current evidence supports MLR as a complementary functional assay rather than a stand-alone clinical decision tool, and standardized monitoring schedules or universally validated implementation pathways are not yet established.

These transplantation-facing applications are summarized in Figure 2, which outlines a conceptual MLR-guided workflow from pre-transplant baseline profiling through early post-transplant monitoring to iterative dose adjustment, with the goal of balancing rejection prevention against infectious and drug-related toxicity. In this framework, multiparametric readouts (e.g., precursor frequency by dye dilution, activation phenotypes, cytokine profiling, and optional TCR-seq tracking) translate donor-reactive signals into functional monitoring outputs. Internationally, related strategies have included flow-based quantification of donor-reactive T cells, cytokine-functional characterization, and TCR-repertoire tracking as complementary approaches to immune-risk assessment in transplantation [94,95,96]. In practice, such functional signals should be interpreted together with routine biomarkers and clinical context rather than used in isolation.

Beyond conventional immunosuppressants, MLR is also used to profile emerging immunomodulators. Studies may leverage MLR to test compounds such as bispecific antibodies that mediate PD-L1-dependent CD28 co-stimulation or to assess how checkpoint-directed agents (e.g., anti-PD-1) reshape T-cell activation, supporting screening of novel immunosuppressive or tolerance-promoting strategies [68]. MLR can additionally flag over-immunosuppression risk by quantifying excessive functional suppression that may predispose to infections or malignancies, helping clinicians balance rejection prevention against toxicity [33].

Recent research also focuses on using MLR to evaluate the impact of immunosuppressants on specific T-cell subsets, such as regulatory T cells (Treg) and Th17 cells whose balance is critical for immune homeostasis and transplant survival. For example, studies might investigate how different immunosuppressants affect the suppressive function of Treg cells or the pro-inflammatory activity of Th17 cells in an MLR setting, providing a more detailed understanding of their immunomodulatory profiles [82,83,97]. The MLR, therefore, remains a vital tool for both preclinical evaluation and clinical monitoring of immunosuppressive therapies, contributing significantly to improved patient and graft survival [33].

Although transplantation remains the most clinically developed application area for MLR, the literature is heterogeneous. Some studies have associated donor-specific hyporesponsiveness with favorable graft outcomes [98], whereas others found that conventional MLR did not predict GvHD severity [99], indicating that predictive value depends on disease context, assay design, readout strategy, and clinical setting. More recent work suggests that donor-specific reactivity can vary substantially across recipient subsets, such as DSA-positive versus DSA-negative kidney transplant recipients [94]. Accordingly, MLR is best interpreted as a context-dependent functional assay that may complement molecular and clinical assessment, rather than as a validated stand-alone predictor of rejection, tolerance, or GvHD [4].

Finally, the MLR has a distinctive role in tolerance research and the mixed-chimerism storyline. In preclinical and translational mixed-chimerism protocols, donor-specific hyporesponsiveness in MLR with preserved third-party responsiveness is widely used as an operational in vitro criterion for tolerance [18,81]. Next-generation integration strengthens this framework: in tolerant human kidney recipients after combined kidney/bone marrow transplantation, MLR-guided identification of donor-reactive clonotypes and subsequent longitudinal tracking provided evidence consistent with clonal deletion of donor-reactive T cells over time [73].

## 7. Applications in Oncology

**Figure 3:**
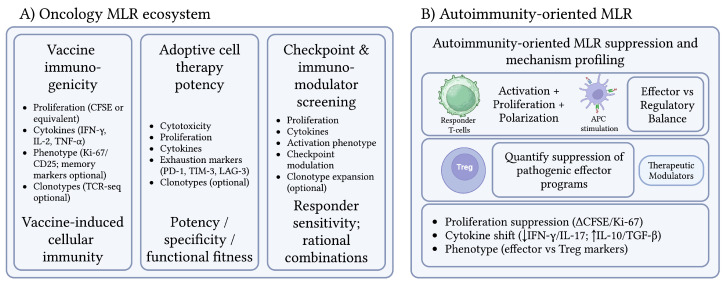
Translational “MLR ecosystem” across in oncology and autoimmunity. (**A**) Oncology “test-bench” applications: MLR-style platforms for vaccine immunogenicity monitoring, adoptive cell therapy potency/specificity testing, and checkpoint/immunomodulator screening using standardized functional outputs (proliferation, cytokines, cytotoxicity, phenotype/exhaustion markers, ± clonotype tracking). (**B**) Autoimmunity-oriented suppression and mechanism profiling: responder T-cell activation and polarization under APC stimulation with addition of regulatory T cells (Treg) and/or therapeutic modulators; suppression is quantified via reduced proliferation and inflammatory cytokine programs, with optional single-cell profiling layers to resolve effector versus regulatory trajectories. Created with BioRender.com.

In oncology, current evidence supports MLR primarily as an ex vivo functional and mechanistic platform for evaluating immunomodulators, dendritic-cell potency, vaccine immunogenicity, and T-cell activation under controlled conditions, rather than as an established clinical predictive assay for patient outcomes [68]. MLR can be used to assess the ability of a patient’s T cells to recognize and respond to tumor-associated antigens (TAAs) or neoantigens. Tumor cells express a variety of antigens, including shared TAAs (present on many tumor types) and patient-specific neoantigens (arising from somatic mutations in individual tumors), both of which can be targets for immune recognition [100]. By co-culturing patient lymphocytes with tumor cells, tumor autologous APCs or genetically engineered APCs expressing specific TAAs or neoantigens, researchers can directly measure the in vitro T-cell response [101]. This involves quantifying T-cell proliferation (e.g., using CFSE dilution), cytokine production (e.g., IFN-γ, IL-2, TNF-α) via ELISA or multiplex assays and, crucially, cytotoxic activity against tumor targets [102]. Conceptually, the same core strength that makes MLR valuable in transplantation—reconstructing a functional immunological synapse in a controlled setting—also enables mechanistic dissection of tumor-reactive immunity and provides a tractable platform to test whether an intervention converts “recognition” into effective effector function. Figure 3A places these oncology applications into a practical “MLR test-bench” structure, spanning vaccine immunogenicity readouts, adoptive cell therapy potency testing, and checkpoint/immunomodulator screening.

This application is particularly useful for evaluating the immunogenicity of cancer vaccines, where the primary goal is to induce robust and durable T-cell responses against tumor antigens to drive anti-tumor immunity in vivo. For peptide-based, dendritic cell-based or viral vector-based vaccines, the MLR serves as a preclinical and early clinical tool to measure the vaccine-induced T-cell responses before and after vaccination [103]. Researchers can assess whether the vaccine successfully generates or expands tumor-specific T cell clonotypes capable of recognizing the target antigens [104].

Functionally, successful priming is reflected by increased T-cell proliferation and effector output, including IFN-γ secretion and/or antigen-specific CTL activity in MLR-based readouts [103]. In oncology monitoring workflows, MLR-style co-cultures are commonly paired with flow cytometry and cytokine profiling to provide a quantitative, phenotype-resolved measure of vaccine-driven responses, including in dendritic-cell vaccine programs [105].

The MLR can also be modified to assess the generation of tumor-specific cytotoxic T lymphocytes (CTLs) that can directly kill cancer cells, which is a critical effector function for successful cancer immunotherapy [102]. MLR-based assays allow for the selection of highly potent, tumor-reactive T cells for expansion and subsequent reinfusion into patients, and the approach aligns naturally with adoptive cell therapy (ACT) workflows in which expanded or engineered T cells must be verified for tumor recognition and killing capacity in vitro [14]. Recent high-throughput adaptations further support this translational use by enabling miniaturized, multiparameter flow cytometry readouts of proliferation, activation, and cytokines in MLR-like formats suitable for screening and optimization [24].

Furthermore, MLR assays are increasingly integrated with advanced technologies like multi-parameter flow cytometry, single-cell RNA sequencing and TCR-sequencing [5,101]. This integration provides deeper insights into the specific tumor-reactive T cell clones, their activation state, differentiation pathways and potential exhaustion markers, which are crucial for understanding resistance mechanisms to therapy [100]. By revealing the functional characteristics of tumor-specific T cells, MLR findings significantly inform the development and refinement of cancer vaccines and adoptive cell therapies, ultimately aiming to improve clinical outcomes [106]. Recent multiparametric profiling of MLR responses has shown that activated proliferating (e.g., Ki-67^+^CD25^+^) responder populations can be linked to distinct cytokine and checkpoint expression patterns, supporting a higher-resolution view of responder quality that is directly relevant when evaluating immunomodulatory interventions [5].

Immune checkpoint molecules (e.g., PD-1, CTLA-4) play a crucial role in suppressing anti-tumor immunity, creating an immunosuppressive tumor microenvironment. MLR assays are extensively used to evaluate the effects of immune checkpoint inhibitors (ICIs) on T-cell activation and proliferation, serving as a powerful preclinical model for drug screening and mechanistic studies [68,83]. The primary mechanism by which PD-1 and CTLA-4 exert their inhibitory effects is by attenuating TCR-mediated positive signaling, leading to reduced proliferation, decreased cytokine secretion (including IL-2 and IFN-γ) and diminished T-cell survival. Specifically, PD-1 engagement recruits phosphatases like SHP-2 to the TCR complex, which dephosphorylate key signaling molecules, thereby inhibiting downstream pathways and IL-2 production [107]. CTLA-4, on the other hand, outcompetes the activating co-stimulatory molecule CD28 for binding to B7 ligands on APCs, delivering an inhibitory signal and limiting T cell activation [108].

When ICIs like anti-PD-1 or anti-CTLA-4 antibodies are introduced into MLR cultures, they block these inhibitory pathways, thereby activating T-cells. This leads to a measurable increase in T-cell proliferation, enhanced production of effector cytokines (such as IL-2 and IFN-γ) and improved cytotoxic activity against target cells [68,101]. In patient-derived settings, in vitro PD-1 blockade has been shown to enhance CD4^+^ and CD8^+^ T-cell function in MLR, including proliferation and IFN-γ production, supporting the relevance of MLR as a functional platform to probe checkpoint responsiveness ex vivo [109].

Because MLR provides a robust and quantifiable readout, it is well-suited for screening novel ICIs, testing rational combinations (including dual PD-1/CTLA-4 blockade), and interrogating resistance mechanisms. For instance, bispecific antibodies that mediate PD-L1-dependent CD28 co-stimulation represent an emerging strategy to augment anti-tumor control in MLR models by coupling checkpoint blockade with active co-stimulation [25].

Furthermore, researchers can utilize MLR to investigate the impact of other immunomodulators, such as silencing PD-L1 or PD-L2 on DCs, which has been shown to enhance proliferation of tumor-specific T cells and increase anti-tumor cytokine secretion, demonstrating the direct impact of these checkpoint molecules on immune responses [44]. MLR can also help elucidate mechanisms of primary and acquired resistance to ICIs by studying the functional characteristics of T cells and APCs from patients who do not respond or relapse after treatment [108]. This involves analyzing changes in gene expression, metabolic profiles or the presence of alternative immune suppressive pathways within the MLR context [110]. The ability to precisely quantify T-cell responses in the presence of various ICIs or their combinations makes the MLR a vital tool for preclinical validation and translational research in the rapidly evolving field of cancer immunotherapy [84,108].

## 8. Role of Mixed Lymphocyte Reaction (MLR) in Understanding Autoimmune Diseases

Autoimmune diseases emerge when immune tolerance fails and lymphocytes mount sustained responses against self-antigens, driving chronic inflammation and organ damage [111,112]. Although the MLR was originally established to model allogeneic T-cell responses, its core value for autoimmunity research lies in the fact that it is a controlled, quantifiable co-culture platform that recreates the same fundamental biology that breaks in autoimmunity: antigen-driven T-cell activation, costimulation, proliferation, and cytokine polarization. In classical descriptions, mixing leukocytes from genetically distinct individuals induces blast transformation, DNA synthesis, and proliferation triggered by recognition of MHC-encoded determinants [113]. This “allostimulation backbone” makes MLR (and MLR-derived suppression formats) a practical experimental scaffold for dissecting effector vs. regulatory balance, mapping inflammatory cytokine programs, and benchmarking how candidate therapies dampen pathogenic T-cell activity or enhance regulation in human cells. These autoimmunity-oriented suppression and mechanism-profiling formats are summarized in Figure 3B, highlighting how MLR-style stimulation can be coupled to regulatory T-cell addition and multiparametric readouts to quantify suppression of pathogenic effector programs.

A major autoimmune-relevant strength of MLR is that it allows subset-resolved functional readouts. Effector differentiation programs that contribute to autoimmunity, particularly Th1 and Th17 polarization, can be inferred from proliferation coupled to cytokine signatures and activation phenotypes. Th17 biology, together with IL-6–dependent pathways, has been emphasized as a key inflammatory axis across immune-mediated disease contexts, and mechanistic work in related immune settings provides a rationale for using MLR-like platforms to quantify Th17-linked activation and cytokine output [114]. Conversely, MLR is widely used in regulatory T-cell (Treg) biology as a functional suppression assay: responder T cells are stimulated (often with irradiated APCs in a MLR format), and graded addition of Tregs quantifies inhibition of proliferation and effector cytokines [115,116]. This is directly aligned with autoimmune pathogenesis, where quantitative and qualitative defects in Tregs (number, stability, suppressive function) are central to loss of tolerance [111,116]. Importantly, newer single-cell-resolved immune profiling approaches can be layered onto MLR-like co-cultures to distinguish proliferating effector vs. regulatory trajectories and to identify functional programs that would be invisible to bulk proliferation measures alone, which is increasingly relevant for mechanism-driven autoimmune therapeutic development [117].

MLR-style assays also provide a useful lens on cytokine “quality,” not just response magnitude. Cytokine skewing toward regulatory profiles (e.g., IL-10/TGF-β-associated programs) versus inflammatory profiles (e.g., IFN-γ, IL-17, IL-6) can be quantified from MLR supernatants or intracellular staining, enabling functional stratification that mirrors clinical heterogeneity in autoimmune disease. A translationally relevant example comes from islet transplantation in type 1 diabetes-related contexts: graft-specific MLC combined with cytokine profiling were associated with clinical outcomes, illustrating how functional cytokine readouts in MLR-type systems can capture immune programs that matter clinically [118]. Finally, because many autoimmune therapies aim either to suppress pathogenic effector T-cell activation or to restore tolerance through regulatory cell augmentation, MLR-based functional assays have remained highly relevant as preclinical-to-translational tools—particularly for evaluating Treg-focused and other regulatory cell-based therapeutic strategies [111,112]. Thus, in autoimmunity, especially in autologous MLR formats, the assay is used mainly to investigate autoreactivity, immune dysregulation, and regulatory-cell function, rather than as a validated routine clinical biomarker across autoimmune diseases [119].

## 9. Limitations of MLR and Practical Implications

Despite its long-standing value as a functional alloreactivity assay, MLR has several methodological and translational constraints that shape how results should be interpreted and compared across studies. At the same time, its major strength is that it captures the integrated biological outcome of donor–recipient cellular interaction, including proliferation, activation, cytokine polarization, and—in newer formats—subset-specific or clonotype-linked response patterns. This gives MLR a distinctive role as a functional complement to molecular matching and other static biomarkers, particularly when the goal is to characterize immune behavior rather than genotype alone. Historically, many protocols relied on ^3^H-thymidine incorporation as a sensitive bulk readout of DNA synthesis, but the requirement for radioisotope handling restricts adoption to appropriately licensed facilities and complicates multicenter harmonization. Non-radioactive, flow-cytometric alternatives (e.g., CFSE for proliferation and PKH-26 for cytotoxicity/CML adaptations) address safety and logistical barriers and can achieve comparability to radiometric approaches, but they still require careful optimization because labeling intensity, toxicity, and species-specific parameters can shift dynamic range and reproducibility (e.g., in large-animal models) [51,55]. In practice, modern flow-based MLR platforms offer important advantages—such as lineage resolution, viability gating, and multiparametric phenotyping—but these benefits come with greater dependence on standardized staining, instrument settings, and analysis pipelines [55,120].

A second limitation is cell-source dependency, especially for stimulator APCs. While DCs are among the most potent stimulators, their scarcity in peripheral blood drives widespread use of monocyte-derived DCs (moDCs) generated in vitro, a practice rooted in the GM-CSF/IL-4 differentiation framework but inherently vulnerable to donor- and protocol-dependent variability (cytokine recipes, maturation stimuli, culture duration, and lot effects) [14,121]. This creates ongoing challenges for standardization and scalability, because the maturation state and cytokine “tone” of DCs can markedly alter priming strength, polarization, and the apparent potency of immunomodulators, complicating cross-study comparisons and clinical translation [14,122]. Accordingly, one practical implication is that MLR results should always be interpreted in light of the cellular configuration used, since differences in APC preparation may reflect technical design choices as much as underlying alloimmune biology. Different moDC maturation strategies are known to generate phenotypically and functionally distinct stimulatory cells, reinforcing the need for protocol transparency and tighter harmonization when comparing studies [123].

Finally, clinical predictive performance is context-dependent. Some cohorts show associations between pre-transplant hyper-responsiveness and outcomes (e.g., kidney graft survival), whereas other settings yield weaker or inconsistent prediction, highlighting that MLR is best interpreted as a probabilistic functional risk signal rather than a deterministic surrogate endpoint [81,124]. This balance of promise and limitation is important in practical terms: MLR may be most useful when deployed as a complementary assay within a broader multimodal framework—for example, alongside graft function, drug exposure, donor-specific antibodies, donor-derived cell-free DNA, biopsy findings, or other immune-monitoring tools—rather than as a stand-alone clinical decision test. Direct validation studies show that flow-based MLR assays can be analytically robust in controlled settings, but broader implementation still requires clearer acceptance criteria, inter-laboratory reproducibility, and prospective validation against meaningful clinical endpoints [74,99].

Taken together, the practical implication of MLR is not that it should replace molecular matching, pharmacokinetic monitoring, or established biomarkers, but that it can add uniquely functional information when a study or clinical question depends on measuring the behavior of donor-reactive immune cells. Its strengths are greatest in mechanistic studies, immunomodulator testing, and selected translational monitoring applications; its limitations become most apparent when assays are insufficiently standardized or when functional signals are overinterpreted as universal predictors across heterogeneous clinical contexts [5,74,99].

## 10. Future Directions to Strengthen Reproducibility and Translational Utility

Several converging trends can mitigate these limitations and expand MLR’s clinical and mechanistic value. First, the field is moving toward assay qualification/validation frameworks borrowed from regulated bioassays—defining precision, linearity/range, and acceptance criteria—particularly when MLR is used as an immunopotency readout for cell therapies or for product comparability across manufacturing changes [74,125]. Second, continued refinement of non-radiolabeled, multiparametric workflows (dye dilution + viability + activation phenotyping + cytokines) should be paired with best-practice guidance on dye selection, labeling conditions, and panel design to reduce technical variance and improve portability across platforms [51,55]. Third, deeper profiling layers—single-cell transcriptomics and clonotype-level TCR tracking—are increasingly positioned to convert MLR from a composite “signal” into interpretable biology (who responds, how they differentiate, and whether specific reactive clones expand or contract over time), which should improve biomarker discovery and mechanistic attribution in transplantation and immune-modulation studies.

A practical future direction is an integrated decision-support framework in which MLR-derived functional alloreactivity is interpreted alongside model-informed precision dosing and adjunct biomarkers, rather than relying on trough concentrations alone. Recent work has highlighted the expanding role of tacrolimus model-informed precision dosing across solid-organ transplantation, including Bayesian forecasting tools, and has also emphasized the potential value of genotype-informed Bayesian approaches, particularly in pediatric recipients, although prospective validation remains limited. In parallel, donor-derived cell-free DNA has emerged as a noninvasive biomarker for kidney allograft rejection, and selective genomic testing has been proposed as part of broader precision-transplant workflows in appropriately selected patients [126,127,128,129,130,131].

In pediatrics, where rejection phenotypes, drug handling, and biomarker performance differ from adults, functional profiling may be especially valuable as part of a multimodal strategy. Recent reviews of pediatric kidney transplantation emphasize that creatinine is a late marker of injury and support combining noninvasive biomarker platforms with more mechanistic immune monitoring to improve early detection and longitudinal risk stratification [132,133,134].

In settings where transplant programs are expanding or where multicenter practice remains heterogeneous, reproducible functional assays and standardized analytics may help improve consistency of immune monitoring and immunosuppression decision-making. This issue is relevant not only for emerging national programs but also for international transplant networks seeking harmonized, comparable functional immune assessment across centers [135,136,137]. Recent consensus statements and multicenter studies in transplantation emphasize that successful implementation of immune-monitoring tools depends on assay standardization, predefined analytical pipelines, and prospective validation across diverse clinical settings. Functional readouts such as donor-reactive T-cell profiling may add complementary information to conventional pharmacokinetic monitoring, but their translational value will depend on robust cross-center reproducibility and harmonized workflows [95,138,139,140].

Finally, the most impactful near-term advance is likely to be protocol harmonization combined with context-specific calibration. In practical terms, this means more consistent reporting and control of core assay variables, including cell sourcing, APC preparation and maturation state, responder:stimulator ratios, culture duration, dye-labeling conditions, gating strategy, and predefined analytical thresholds. Experience from flow-cytometry standardization and assay-validation literature shows that such measures are essential to improve reproducibility, reduce inter-laboratory variability, and support portability across platforms. In parallel, context-specific calibration remains important because the intended use of MLR differs across applications: mechanistic studies may prioritize phenotypic depth, potency assays may require defined precision, linearity, and release criteria, whereas translational monitoring may require stable longitudinal comparability and integration with clinical covariates [51,55,74,125].

Accordingly, future progress will depend not only on adding more complex readouts, but also on building clearer implementation frameworks that specify intended use, minimum reporting elements, shared controls or donor pools where appropriate, and acceptance criteria tailored to the biological and translational question. This approach is consistent with broader biomarker and immune-monitoring efforts in transplantation, where recent consensus work emphasizes that successful clinical implementation requires assay standardization, predefined analytical pipelines, and prospective validation across centers and patient populations [138,139,140]. In this sense, MLR is best viewed as a modular functional assay with strongest translational maturity in transplantation and substantial value as a mechanistic and assay-development platform in oncology, autoimmunity, and cell-therapy research. Flow cytometry standardization work shows that consistent sample handling, reagent selection, instrument setup, and analysis rules are major determinants of reproducibility across laboratories [120,141]. Recent transplant-biomarker consensus work likewise stresses that implementation requires predefined analytical pipelines and prospective multicenter validation rather than isolated single-center performance [138,142].

## 11. Conclusions

Modern mixed lymphocyte reaction (MLR) platforms have moved well beyond a legacy “bulk proliferation test” and now function as a modular experimental ecosystem that couples a controllable allogeneic stimulus to increasingly precise, scalable readouts. As summarized in the current draft, this evolution positions MLR as a multidimensional immune-profiling tool that captures integrated effects of allorecognition, co-stimulation, inflammatory context, and minor histocompatibility determinants that are not always inferable from molecular matching alone. However, predictive performance remains context- and protocol-dependent. In practice, this means that the clinical relevance of an MLR result may differ according to transplant type, immune-risk subgroup, assay design, readout strategy, and the extent to which the assay is interpreted alongside other clinical and biomarker data. Accordingly, MLR is best viewed as a probabilistic functional signal rather than a stand-alone surrogate endpoint.

Across contemporary designs, the core strength of MLR remains its ability to link assay architecture (one-way vs. two-way format; stimulator inactivation; responder definition; APC source and maturation state) to interpretable biological questions, including rejection/GvHD risk modeling, tolerance-oriented studies, and immunomodulatory screening. This review highlights how radiometric, flow cytometric (dye dilution and Ki-67), cytokine/chemokine profiling, cytotoxicity adaptations, and next-generation integrations (e.g., scRNA-seq and TCR sequencing) provide complementary “views” of alloreactivity, allowing investigators to choose the resolution and mechanism needed for a given translational aim.

Looking forward, broader adoption of MLR in high-grade translational research will depend less on inventing new readouts and more on harmonizing study design and validation-oriented quality controls. Treating MLR as a quasi-quantitative bioassay—explicitly managing donor variability, dynamic range, timing, batch effects, and acceptance criteria—can substantially improve reproducibility and cross-study comparability, strengthening the assay’s utility for clinically meaningful immune monitoring and for biomarker-guided personalization in transplantation, immunotherapy, and immune-modulation research.

## Figures and Tables

**Table 1:** Cell sources and APC configurations in MLR assays: implications for readouts and interpretation.

Design Element	Typical Options/ Implementation	What It Captures (Strengths)	Limitations/ Caveats	References
Starting cell source	PBMCs as default for responders and/or stimulators (density gradient isolation).	Readily accessible; contains responder T-cell pool plus physiologic APC mixture; robust proliferation/cytokine output due to high alloreactive precursor frequency.	Composite biology; APC composition varies across donors; less mechanistic control	[34]
Responder compartment definition	Whole PBMC responders vs. purified T-cell subsets (e.g., CD3^+^, CD4^+^, CD8^+^, memory subsets via MACS/FACS).	Purified responders increase analytical resolution and allow lineage-specific interpretation of proliferation and function.	Additional processing; subset purity/activation during isolation can affect baseline.	[43,44]
Core activation requirements	Allorecognition-driven activation requires TCR-MHC engagement, costimulation, and cytokine programming.	Provides mechanistic framework linking APC phenotype to responder expansion, differentiation, and effector function.	Context-dependent; sensitive to culture conditions and APC maturation state.	[11,44]
CD4^+^ T-cell contribution	CD4^+^ recognize alloantigen on MHC class II; differentiate into effector/regulatory programs (Th1/Th2/Th17/Treg).	Orchestrates alloresponse; supports CD8^+^ activation; shapes cytokine milieu and regulatory balance.	Subset balance varies with APC type and maturation; interpretation benefits from multiparametric readouts.	[11,44]
CD8^+^ T-cell contribution	CD8^+^ CTLs recognize alloantigen on MHC class I; often require CD4^+^ help/cytokines for full activation.	Links MLR to cytotoxic effector pathways relevant to graft injury.	CD8^+^ responses may be underestimated in bulk assays without phenotype-resolved readouts.	[11,43,44]
Single-cell profiling in MLR	scRNA-seq/single-cell approaches applied to proliferating responders.	Reveals heterogeneous, polyfunctional proliferating CD4^+^/CD8^+^ states and distinct activation programs.	Cost/complexity; requires careful experimental design to connect states to function.	[5]
Stimulator APC source	Whole PBMC stimulators (monocytes/B cells as major APCs) vs. DC-enriched systems.	PBMC stimulators are pragmatic/physiologic; DC-enriched systems yield stronger, more controllable allostimulation.	PBMC APC fraction varies in abundance/maturation; PBMC APCs may be weaker stimulators than DCs.	[13]
Use of moDCs (common DC-enriched approach)	Differentiate moDCs from CD14^+^ monocytes ex vivo to overcome low circulating DC frequency.	Potent stimulation; consistent APC source; compatible with mechanistic and immunomodulation studies.	Differentiation protocols vary; phenotype may differ from primary DC subsets.	[13,44]
DC maturation state (experimental “dial”)	Mature DCs (inflammatory maturation cues) vs. immature/tolerogenic DCs (e.g., IL-10/TGF-β-conditioned).	Mature DCs drive strong expansion and polyfunctional cytokines; tolerogenic DCs bias toward hyporesponsiveness/anergy/Treg induction.	Maturation status must be validated; outcomes can be protocol-specific.	[45,46]
APC diversity beyond moDCs	cDC1, cDC2, mcDCs; experimental use of specific APC subsets.	Enables testing how APC metabolic/functional programs shape T-cell outcomes.	Access/rarity; isolation and maturation standardization can be challenging.	[13,47]
pDCs in MLR/transplant contexts	pDCs generally weaker APCs; may influence Th17-associated pathways in some clinical contexts.	Relevant where pDC-linked pathways are biologically central (e.g., certain GvHD-associated immune programs).	Usually not primary drivers of classical MLR proliferation; interpret carefully.	[47]
NK cells (context-dependent)	NK cells present in PBMCs; may be depleted for T cell-centric designs.	Can contribute under “missing-self” conditions and in GvHD/cell therapy-relevant biology.	Often dispensable for initiating canonical T-cell-driven MLR; inclusion should match study goal.	[12]

Abbreviations: APC, antigen-presenting cell; cDC1/cDC2, conventional dendritic cell subset 1/2; CTL, cytotoxic T lymphocyte; DC, dendritic cell; FACS, fluorescence-activated cell sorting; GvHD, graft-versus-host disease; IL, interleukin; MACS, magnetic-activated cell sorting; mcDC, merocytic dendritic cell; MHC, major histocompatibility complex; MLR, mixed leukocyte reaction; moDC, monocyte-derived dendritic cell; NK, natural killer (cell); PBMC, peripheral blood mononuclear cell; pDC, plasmacytoid dendritic cell; scRNA-seq, single-cell RNA sequencing; TCR, T-cell receptor; TGF-β, transforming growth factor beta; Th, T helper (cell); Treg, regulatory T (cell).

**Table 2:** Methods to Measure Outcomes of Mixed Lymphocyte Reaction (MLR).

Method	Description	References
Flow Cytometry and Differential Gating	Analyzes cell populations based on scatter properties and fluorescence microspheres.	[50]
CFSE Labeling	Tracks cell proliferation and cytokine secretion using CFSE and ICIS.	[16,51]
Multiparametric Flow Cytometry	Simultaneous determination of proliferation and cytokine activity.	[5,16,24]
Vital Dye Labeling	Uses CFDA-SE and SNARF-1 dyes for detailed proliferation analysis.	[52]
Multiplex Cytokine Assays	Measures cytokine and chemokine secretion, combined with RNA sequencing.	[5]
Radioisotope Labeling	Traditional method using ^3^H-thymidine incorporation for DNA synthesis.	[50,51,53]
CCK-8 Assay	Measures cellular proliferation through cell counting and CCK-8 assay.	[54]
High-throughput Flow Cytometry	Advanced platform for immunophenotyping and screening multiple readouts.	[24]

Abbreviations: MLR, Mixed Lymphocyte Reaction; CFSE, carboxyfluorescein diacetate succinimidyl ester; ICIS, intracellular cytokine staining; CFDA-SE, carboxyfluorescein diacetate succinimidyl ester; SNARF-1, seminaphthorhodafluor-1; ^3^H-thymidine, tritiated thymidine; CCK-8, Cell Counting Kit-8.

**Table 3:** Outcome readouts in MLR: what they measure, strengths, and practical limitations.

Readout Domain	Method (Examples)	What it Measures in MLR	Key Strengths	Key Limitations/ Pitfalls	References
Proliferation	Radiometric DNA synthesis: ^3^H-thymidine incorporation	Bulk DNA synthesis (S-phase entry) as a surrogate of total proliferation.	Highly sensitive; long-standing benchmark; simple quantitative output.	No subset resolution; radioisotope handling/disposal; timing-sensitive labeling window; radiotoxicity can perturb cell cycle.	[50,53,59]
	Dye-dilution flow cytometry: CFSE/CFDA-SE, CellTrace Violet, CellTrace Far Red.	Division history at single-cell level; proliferation index/precursor frequency; subset-specific expansion.	Resolves multiple generations; compatible with multiparametric phenotyping.	Dye toxicity/perturbation at high concentration; compensation/spectral spillover; peak compression if staining suboptimal.	[16,22,24,48,50,55,60]
	Cell-cycle/proliferation marker: Ki-67 (flow cytometry).	Growth fraction (cells in G1/S/G2/M; absent in G0); proliferating responder subsets.	Sensitive, stable readout; useful when dye dilution is limited or subtle responses are expected.	Requires fixation/permeabilization; kinetics differ from dye dilution; interpretation depends on culture duration.	[5,53,61]
Activation phenotype and viability	Activation markers: CD69 (early), CD25 (IL-2Rα), HLA-DR, plus subset gating (CD4/CD8; naïve/memory).	Activation state of responding T cells; lineage-resolved response dynamics.	Adds mechanistic resolution beyond “how much proliferation”; enables responder compartment attribution.	Marker expression is time-dependent; activation can occur without proliferation; requires consistent gating strategy.	[5,16,62]
	Survival/apoptosis: Annexin V, 7-AAD/PI, live/dead dyes	Distinguishes viable proliferating responders from dying/bystander cells.	Prevents false interpretation from differential survival; improves signal-to-noise.	Staining order and timing matter; apoptosis can be culture-condition dependent.	[16,50]
Cytokines and chemokines	Single-analyte: ELISA.	Concentration of individual cytokines (e.g., IFN-γ, IL-2, IL-10) in supernatant.	Straightforward; clinically familiar; good quantitative performance per analyte.	Limited multiplexing; larger sample volumes across panels.	[5,63,64,65]
	Multiplex bead-based assays: Luminex/xMAP, Cytometric Bead Array (CBA)	Multi-cytokine/chemokine “palette” defining response skewing (Th1/Tc1 vs. Th2/Tc2 vs. Th17 vs. Treg-associated patterns).	High content from small volumes; scalable; supports pathway-level interpretation.	Cross-reactivity/standardization issues; platform-specific dynamic ranges; careful QC required.	[24]
Cytotoxicity	Cell-mediated lympholysis (CML)/^51^Cr release.	Effector killing capacity of MLR-primed CTLs against labeled targets.	Direct functional cytotoxicity endpoint; classical CTL assay paired with MLR priming.	Radioisotope handling; requires optimized E:T ratios; may not reflect in vivo microenvironment.	[51]
	Flow/fluorescent target killing (e.g., PKH-26 labeling; antigen/lineage markers) or bioluminescent targets (luciferase).	Target-specific killing without radioactivity; adaptable to tumor-target co-culture models.	Compatible with multiparametric phenotyping; scalable and safer than ^51^Cr.	Assay design variability; requires rigorous controls for target loss vs. death	[14]
Next-generation integrations	scRNA-seq/transcriptomics (single-cell or bulk).	Transcriptional programs of responding subsets; pathway activation (e.g., NF-κB, JAK/STAT); discovery of biomarkers of reactivity.	Mechanistic depth; identifies responder states and regulatory checkpoints; integrates with cytokine/proliferation phenotypes.	Cost; bioinformatics burden; batch effects; careful experimental design required.	[5]
	TCR sequencing coupled to MLR enrichment.	Donor-reactive clonotype “fingerprint”; longitudinal tracking for clonal expansion (rejection) vs. deletion (tolerance).	Personalized tracking beyond bulk function; supports immune monitoring without repeating functional assays.	Requires baseline donor–recipient MLR sequencing; interpretation depends on sampling depth and repertoire dynamics	[4]

Abbreviations: MLR, Mixed Lymphocyte Reaction; ^3^H-thymidine, tritiated thymidine; CFSE, carboxyfluorescein diacetate succinimidyl ester; CFDA-SE, carboxyfluorescein diacetate succinimidyl ester; HLA-DR, human leukocyte antigen–DR; IL-2Rα, interleukin-2 receptor alpha; 7-AAD, 7-aminoactinomycin D; PI, propidium iodide; ELISA, enzyme-linked immunosorbent assay; IFN-γ, interferon gamma; IL, interleukin; Th, T helper; Tc, cytotoxic T; Treg, regulatory T; xMAP, multi-analyte profiling (Luminex xMAP technology); CBA, cytometric bead array; CML, cell-mediated lympholysis; CTL, cytotoxic T lymphocyte; ^51^Cr, chromium-51; E:T, effector-to-target ratio; PKH-26, PKH26 fluorescent membrane dye; scRNA-seq, single-cell RNA sequencing; NF-κB, nuclear factor kappa B; JAK/STAT, Janus kinase/signal transducer and activator of transcription; TCR, T-cell receptor.

**Table 4:** Quality and reproducibility controls for Mixed Lymphocyte Reaction (MLR) assays.

Quality Domain	Key Recommendation (Checklist)	Why It Matters	Typical Implementation/Notes	Evidence	Reference
Donor variability management	Equitably distribute responders/stimulators from the same donor pools across arms.	Inter-donor biology is often the dominant variance component.	Randomize plate layout; keep donor pairing constant within comparisons.	mdMLR and potency work highlights donor/pool variability as a key driver of assay spread.	[66]
	Use multidonor pools to stabilize the stimulatory signal (QC reagent)	Pooling can reduce variance and increase signal consistency.	Pools of 5–8 donors used as standardized stimulators in some potency-style designs.	Historical analysis supports pooled targets to increase magnitude and reduce variation; modern pooled-PBMC stimulator workflows used in MLR studies.	[67,68]
	In humanized models, use animals generated from the same cord blood batch.	Reduces genetics/engraftment-kinetics confounding.	Batch-restricted cohorts; consistent gating/absolute quantitation.	Modeling best practice; include if your review covers in vivo–ex vivo integration.	[69]
	Track expansion using absolute counts, not only percentages.	Percentages can be distorted by changing background populations or gating.	Report cells/µL or total recovered responders per well.	Aligns with general flow cytometry quantitation principles)	[70]
Responder: Stimulator ratios (dynamic range)	Use standard APC:T-cell ratios (e.g., 1:10) for baseline activation.	Ratio determines assay sensitivity, peak response, and nutritional stress.	Optimize per APC type (PBMC vs. DC) and responder subset.	Early work shows peak depends on target/responder ratio; many protocols use 1:1 to 1:4 for peak in classic MLR contexts.	[50,67,71]
	Titrate stimulator potency for highly immunogenic stimulators (avoid overstimulation).	Overstimulation can mask inhibition and reduce viability.	Lower stimulator density or shorten culture to preserve discrimination.	Kinetic CFSE study shows division dynamics shift with time/conditions; supports need to tune culture parameters.	[72,73]
	For immunosuppression testing, use a ratio series (e.g., 1:1 → 1:0.1) to define discriminatory range.	Needed to detect batch differences and define “limit of detection” for inhibition.	Choose one “most sensitive” ratio as release-discriminator once established.	Potency-oriented MLR validation frameworks emphasize defined range/linearity for product testing.	[64,74]
Culture duration and timing	Use defined readout windows (proliferation often detectable ~day 5–8).	Proliferation kinetics vary; late timepoints confound survival/apoptosis.	Classic microculture MLR often runs ~6–7 days.	Overview sources describe 6–7-day incubation for primary one-way MLR microcultures.	[14,64,75]
	For dye-dilution flow, select timepoints before peak overlap/decay (e.g., day ~4–6 depending on dye).	Late culture increases autofluorescence/peak compression + death.	Pilot a time-course per dye and platform.	CFSE/CTV division kinetics in MLR show strong changes across days 2–7; emphasizes time-course optimization.	[72]
	Standardize dye labeling (fixed incubation time).	Small variations alter baseline intensity → mis-called generations.	Use standard operating procedures (SOPs) and QCs per batch.	Dye dilution method guidance emphasizes protocol standardization and technical pitfalls.	[51,55]
Batch effects and standardization	Use standardized polyclonal stimulation (e.g., anti-CD3/CD28) to reduce variability.	Increases reproducibility when alloreactivity is weak/variable.	Beads or plate-bound formats; keep CD3:CD28 density consistent.	Anti-CD3/CD28 stimulation established to reliably activate/expand human T cells; bead coating ratios affect response.	[64,76]
	Prefer robust markers for diagnostics/QC (e.g., Ki-67).	Less sensitive to dye toxicity and some handling variability.	Combine with lineage gating (CD4/CD8) and activation markers.	Flow-MLR validation and immune profiling studies commonly incorporate Ki-67 for proliferation state.	[53,64,74]
	Remove dead cells/apply viability dyes (e.g., 7-AAD, platinum-based dyes).	Dead/apoptotic cells increase nonspecific binding and artifacts.	Include live/dead gate; consider cleanup if death is high.	Cisplatin viability labeling validated for single-cell cytometry discrimination of live/dead.	[64,77]
Endpoint harmonization and acceptance criteria	Define minimum positive-control responsiveness (e.g., ≥5 daughter generations).	Ensures responders were competent and the assay ran in-range.	Establish per platform/dye; document in SOP.	Dye-dilution kinetics show generation structure and timing are assay-dependent; supports explicit generation-based criteria.	[55,64,72]
	Set analysis quantitation limits (e.g., minimum events per generation cluster).	Prevents over-calling noise as true proliferation peaks.	Predefine LLOQ (events) and gating templates.	Dye-dilution best-practice papers emphasize rigorous gating and QC to avoid misassignment of generations.	[64]
	For clinical batch release, specify inhibition thresholds (e.g., ≥40% inhibition at 1:1).	Converts assay output into actionable release criteria.	Thresholds should be product- and lab-validated.	Potency assay validation for MSCs/EVs discusses assay qualification and need for defined acceptance criteria/range.	[64,66,74]
	For cytotoxicity adaptations, subtract nonspecific background.	Controls for spontaneous target death/label leakage.	Include target-only controls; background-correct all readouts.	Standard principle for cytotoxicity readouts.	[51]

**Table 5:** Summary of MLR Clinical and Biological Applications.

Clinical/Biological Context	Typical MLR Design	Primary Readouts	Decision/Insight Generated	Representative Biomarkers/Signatures	References
Solid Organ Transplantation	One-way MLR: Recipient T cells (responders) + Inactivated Donor PBMC or moDC (stimulators)	Multiparametric flow (CFSE/VPD450); TCR-seq to map the donor-reactive repertoire	Functional assessment of donor-reactive alloresponse; Monitoring for donor-specific hyporesponsiveness to guide immunosuppression tapering	SI (Stimulation Index); Donor-reactive clonotypes (fingerprinting); HLA-DP reactivity	[14,51]
Hematopoietic Stem Cell Transplant	One-way MLC: Cytokine-spiked (IL-2, IL-4, IFN-γ, TNF-α) to increase sensitivity	Proliferation (^3^H-thymidine); TCR-seq; Multiplex cytokines (CBA)	Modeling of donor-versus-recipient alloreactivity relevant to GvHD (especially in HLA-identical siblings); Assessment of Graft-versus-Leukemia (GVL) potential	SI > 1 in modified MLC; IL-10 spot-forming cells (associated with chronic GvHD); Th17/Th1 skewing	[4,29]
Cancer Vaccines	Antigen-loaded APC: Autologous T cells Patient-derived moDCs loaded with tumor antigens (TSA) or lysates	Proliferation; Activation phenotype (flow); Soluble cytokines	Evaluation of vaccine immunogenicity; Identification of most potent tumor-specific antigen (TSA) candidates	IFN-γ, IL-2, and TNF-α secretion; CD25^+^ CD69^+^ activation phenotype	[14]
Adoptive Cell Therapy (ACT)	Tumor-target co-culture: Effector cells (e.g., chimeric antigen receptor T-cell, CAR-T) luciferase-expressing or fluorescently labeled tumor lines	Cytotoxicity (Bioluminescence, PKH-26); scRNA-seq for clonal tracking	ACT potency and tumor-killing efficacy; Predicting risk of CAR-T rejection or GvHD in allogeneic contexts	Granzyme B/Perforin; CAR-T expansion; Pro-inflammatory signatures (e.g., IL-6)	[14,51]
Checkpoint Blockade	High-throughput MLR: Miniaturized (e.g., 384-well platform) screening for compound libraries	Multiparametric flow (Ki-67/CD25); scRNA-seq for mechanistic signaling	Functional evaluation of checkpoint-modulated T-cell activation; Insights into the abrogation of immune suppression	Ki-67+ CD25+ immunoreactive clusters; Expression of PD-1, TIM-3, LAG-3; PBK, LRR1, MYO1G	[5,24]
Autoimmunity and Treg Therapy	Suppression format: One-way MLR + candidate suppressive cells (Tregs, MSCs, or MSC-EVs)	Inhibition index; Cytokines (IL-10, TGF-β); scRNA-seq	Evaluation of effector-regulatory balance; Quality control release criteria for clinical cell products	FoxP3^+^ stability; IL-10/TGF-β anti-inflammatory profile; miR-638/Fosb axis	[64,65]

## Data Availability

No new data were created or analyzed in this study.

## Abbreviations

The following abbreviations are used in this manuscript:

ACT	Adoptive cell therapy
APC	Antigen-presenting cell
CBA	Cytometric bead array
CCK-8	Cell Counting Kit-8
cDC1	Conventional dendritic cell subset 1
cDC2	Conventional dendritic cell subset 2
CFDA-SE	Carboxyfluorescein diacetate succinimidyl ester
CFSE	Carboxyfluorescein succinimidyl ester
CML	Cell-mediated lympholysis
CTL	Cytotoxic T lymphocyte
DC	Dendritic cell
dd-cfDNA	donor-derived cell-free DNA
ELISA	Enzyme-linked immunosorbent assay
EV	Extracellular vesicle
GM-CSF	Granulocyte–macrophage colony-stimulating factor
GvHD	Graft-versus-host disease
HSCT	Hematopoietic stem cell transplantation
ICI	Immune checkpoint inhibitor
iPSC	Induced pluripotent stem cell
LLOQ	Lower limit of quantitation
MHC	Major histocompatibility complex
miHA	Minor histocompatibility antigen
MLR	Mixed lymphocyte reaction
MMC	Mitomycin C
moDC	Monocyte-derived dendritic cell
MSC	Mesenchymal stromal/stem cell
NF-κB	Nuclear factor kappa B
NK	Natural killer (cell)
PBMC	Peripheral blood mononuclear cell
pDC	Plasmacytoid dendritic cell
PI	Propidium iodide
PKH-26	PKH26 membrane dye
scRNA-seq	Single-cell RNA sequencing
SHP-2	Src homology 2 domain-containing phosphatase-2
SI	Stimulation index
SNARF-1	Seminaphtharhodafluor-1
TAA	Tumor-associated antigen
TCR	T cell receptor
TSA	Tumor-specific antigen
VPD450	Violet proliferation dye 450
